# Sparse CCA-based mediation analysis with high-dimensional exposures and mediators

**DOI:** 10.1093/bioinformatics/btag474

**Published:** 2026-06-30

**Authors:** Xincheng Li, Maiying Kong, Matthew Ryan Smith, Yongliang Liang, Sami Teeny, Vilinh T Ly, Young-Mi Go, Niharika Samala, Dean P Jones, Jianzhu Luo, Walter H Watson, Craig J McClain, Vatsalya Vatsalya, Gyongyi Szabo, Srinivasan Dasarathy, Mack Mitchell, Laura E Nagy, Bruce Barton, Matthew C Cave, Hongmei Jiang

**Affiliations:** Department of Statistics and Data Science, Northwestern University, Evanston, IL 60208, United States; Department of Bioinformatics and Biostatistics, University of Louisville, Louisville, KY 40202, United States; VA Healthcare System of Atlanta, Decatur, GA 30033, United States; Division of Pulmonary, Allergy, Critical Care and Sleep Medicine, Department of Medicine, Emory University School of Medicine, Atlanta, GA 30322, United States; Division of Pulmonary, Allergy, Critical Care and Sleep Medicine, Department of Medicine, Emory University School of Medicine, Atlanta, GA 30322, United States; Division of Pulmonary, Allergy, Critical Care and Sleep Medicine, Department of Medicine, Emory University School of Medicine, Atlanta, GA 30322, United States; Division of Pulmonary, Allergy, Critical Care and Sleep Medicine, Department of Medicine, Emory University School of Medicine, Atlanta, GA 30322, United States; Division of Pulmonary, Allergy, Critical Care and Sleep Medicine, Department of Medicine, Emory University School of Medicine, Atlanta, GA 30322, United States; School of Medicine, Indiana University, Indianapolis, IN 46202, United States; Division of Pulmonary, Allergy, Critical Care and Sleep Medicine, Department of Medicine, Emory University School of Medicine, Atlanta, GA 30322, United States; School of Medicine, University of Louisville, Louisville, KY 40202, United States; School of Medicine, University of Louisville, Louisville, KY 40202, United States; School of Medicine, University of Louisville, Louisville, KY 40202, United States; Robley Rex VA Medical Center, VA Louisville Healthcare System, Louisville, KY 40206, United States; School of Medicine, University of Louisville, Louisville, KY 40202, United States; Beth Israel Deaconess Medical Center, Harvard Medical School, Boston, MA 02215, United States; Cleveland Clinic, Cleveland, OH 44195, United States; University of Texas Southwestern Medical Center, Dallas, TX 75390, United States; Cleveland Clinic, Cleveland, OH 44195, United States; Department of Population and Quantitative Health Sciences, University of Massachusetts, Worcester, MA 01655, United States; School of Medicine, University of Louisville, Louisville, KY 40202, United States; Robley Rex VA Medical Center, VA Louisville Healthcare System, Louisville, KY 40206, United States; Department of Statistics and Data Science, Northwestern University, Evanston, IL 60208, United States

## Abstract

**Motivation:**

Mediation analysis plays a crucial role in understanding how exposure variables influence health outcomes via intermediate variables, or mediators, in environmental studies. When analysing a large number of environmental exposures, such as chemical mixtures or pollutants, together with multiple potential mediators such as metabolites, advanced methodologies are necessary to accurately separate direct and indirect effects. This paper proposes a novel mediation analysis method based on Sparse Canonical Correlation Analysis (SCCA), designed specifically for settings where both exposures and mediators are high-dimensional. The effectiveness of the proposed method is evaluated through simulation studies and an application to real-world data.

**Results:**

The proposed SCCA-based mediation framework improved identification of relevant mediators and pathways in simulation studies, particularly in high-dimensional and noisy settings. The two-step screening extension further enhanced feature selection while maintaining stable estimation. In the real-data application, the method identified interpretable exposure–metabolite pathways associated with MELD score, with several pathways showing moderate selection stability and robustness to potential unmeasured confounding.

**Availability:**

The R code for implementing the proposed method and the simulation studies is available at https://github.com/MaggieLi2001/HDM-SCCA2.

## 1 Introduction

Mediation analysis is a fundamental statistical framework for examining how an exposure is associated with an outcome through an intermediate variable, referred to as a mediator. The seminal work of Baron and Kenny (1986) formalized mediation analysis using a system of regression models that impose a structured ordering among the exposure, mediator, and outcome. By decomposing the total effect into direct and indirect components, mediation analysis provides a useful tool for characterizing intermediate pathways. Several estimation strategies have been proposed, including the causal steps approach, the difference-in-coefficients method, and the product-of-coefficients method (MacKinnon *et al.* 2007), all of which rely on regression-based formulations linking the exposure, mediator, and outcome (Rijnhart *et al.* 2021). Traditional approaches, such as the Baron–Kenny framework and classical structural equation modeling (SEM) (Fiske *et al.* 1982), are primarily developed for low-dimensional settings and do not readily scale to applications involving high-dimensional mediators or exposures (Gunzler *et al.* 2013). Although SEM allows for simultaneous estimation and latent variable modeling, standard implementations remain limited in high-dimensional mediation contexts.

Recent methodological developments have focused on mediation analysis with a single exposure and multiple high-dimensional mediators MacKinnon (2000). Among these, High-Dimensional Mediation Analysis (HIMA) Zhang *et al.* (2016) and its extension HIMA2 Perera *et al.* (2022) combine screening and penalized regression techniques to identify relevant mediators. HIMA employs sure independence screening (SIS) Fan and Lv (2008) and minimax concave penalty (MCP) regression with Bonferroni-corrected joint significance testing Zhang *et al.* (2016); Zhang (2010), while HIMA2 improves efficiency by incorporating indirect effects into screening, using de-biased Lasso estimation Tibshirani (1996), and adopting FDR-controlled inference Perera *et al.* (2022). However, these methods are primarily designed for settings with low-dimensional exposures. In applications such as multi-omics, neuroimaging, and environmental health studies, both exposures and mediators are often high-dimensional, and strong correlations among variables pose additional challenges (Nath *et al.* 2023). Dimension-reduction approaches based on principal component analysis (PCA) and sparse PCA (SPCA) mitigate multicollinearity and improve interpretability (Zhao *et al.* 2020, Zhao and Li 2022), but they largely focus on within-block structure and do not explicitly model exposure–mediator dependence.

Motivated by these limitations and a real-world environmental health research where both high-dimensional environmental exposures and high-dimensional metabolites acting as mediators influence health outcomes Dasarathy *et al.* (2020), we propose a mediation framework based on sparse canonical correlation analysis (SCCA) (Hardoon and Shawe-Taylor 2011, Wilms and Croux 2016). SCCA performs joint dimension reduction and feature selection by identifying sparse linear combinations of exposures and mediators that maximize their correlation, thereby enhancing interpretability and addressing multicollinearity. We refer to this approach as high-dimensional mediation analysis via SCCA (HDM-SCCA). In this paper, we present the proposed mediation analysis, focusing on its application to settings with high-dimensional exposures, high-dimensional mediators, and a continuous numerical outcome. In Section 2, we introduce the proposed method, outlining its theoretical foundations and implementation. Section 3 presents simulation studies to evaluate the performance of the method in various high-dimensional scenarios. In Section 4, we apply this model to a real-world data. Finally, Section 5 concludes with a summary of findings and potential future directions.

## 2 HDM-SCCA mediation model

In this study, we consider a high-dimensional mediation analysis framework where both exposure variables and mediators are high-dimensional under standard linear mediation assumptions. Specifically, we assume linear and additive relationships among exposures, mediators, and outcomes. We further assume there is no unmeasured confounding for the exposure–mediator, mediator–outcome, and exposure–outcome relationships after adjustment for observed covariates. No exposure-mediator interaction is assumed. We further assume the absence of causal relationships among individual mediator processes.

Let $E_{1},\ldots ,E_{p}$ denote the exposure variables and define the random exposure vector as $E_{\text{var}}={\left(E_{1},\ldots ,E_{p}\right)}^{⊤}\in R^{p},$ which consists of the *p* exposure variables. $E\in R^{p\times n}$ denotes the data matrix with *p* exposures (rows) and *n* subjects (columns). Similarly, let $M_{1},\ldots ,M_{q}$ denote the *q* mediator variables, and define the random mediator vector as $M_{\text{var}}={\left(M_{1},\ldots ,M_{q}\right)}^{⊤}\in R^{q}.$  $M\in R^{q\times n}$ denotes the data matrix with the same *n* subjects and *q* mediators. Let *Y* denote the random outcome variable of interest. For the *n* subjects, the observed outcome vector is $Y=(Y_{1},Y_{2},\ldots ,Y_{n})\in R^{n\times 1}$. It is associated directly with the exposures and indirectly through the mediators. In real-world applications, both the number of exposures and the number of mediators can be high dimensional, while only a small subset of these variables are truly associated with the outcome. Analysing such high-dimensional data presents significant challenges, as the presence of many noisy variables can increase the risk of overfitting, reduce interpretability, and lead to spurious associations.

**Figure 1:**
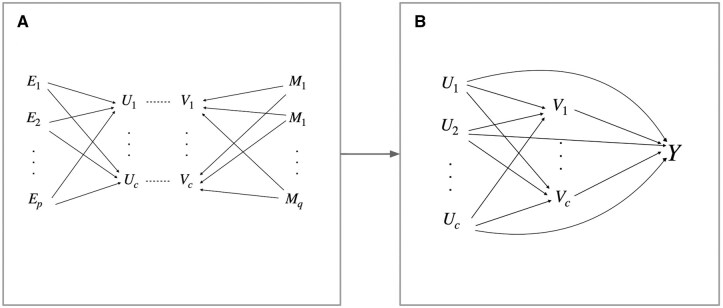
Workflow of HDM-SCCA: (A) High dimensional exposures ($E_{1},\ldots ,E_{p}$) and mediators ($M_{1},\ldots ,M_{q}$) are mapped to their canonical representations ($U_{k}$, $V_{k}$), k = 1, …c, using SCCA, where c is the optimal number of canonical pairs. (B) Mediation analysis is performed on the canonical variables.

To address these challenges in mediation analysis with high-dimensional exposures and mediators, we propose a new high-dimensional mediation method based on SCCA, which denotes as HDM-SCCA (Fig. 1). First, SCCA is performed on the exposure variables and mediator variables, and subsets of sparse canonical components are selected. Then, mediation analysis is performed on these canonical components, and the direct and indirect effects are estimated. To assess whether the feature screening step improves the performance of the HDM-SCCA framework, we also evaluate a modified approach, HDM-SCCA2, which incorporate a two-step Elastic Net procedure for feature selection before applying HDM-SCCA in simulation studies (Zou and Hastie 2005, Tay *et al.* 2023).

### 2.1 Sparse CCA

Canonical Correlation Analysis (CCA) is used to handle two sets of variables by identifying linear combinations of variables in the two sets that are maximally correlated. These canonical variables capture the strongest associations between exposures and mediators (Witten *et al.* 2009). SCCA is an extension of CCA that introduces sparsity constraints to mitigate overfitting and enhance interpretability. This is particularly useful in situations where the number of variables exceeds the sample size, a scenario where traditional CCA tends to struggle. SCCA selects a subset of relevant variables from each set, ensuring that only the most important features contribute to the canonical variates, thus improving model efficiency and reducing redundancy.

The goal of SCCA is to extract pairs of canonical variables $\left(U_{k},V_{k}\right)$ for k=1,…,min(p,q), where $U_{k}$ is derived from the exposure vector $E_{\text{var}}={\left(E_{1},\ldots ,E_{p}\right)}^{⊤}$ and $V_{k}$ is derived from the mediator vector $M_{\text{var}}={\left(M_{1},\ldots ,M_{q}\right)}^{⊤}$. Each canonical variable is expressed as a linear combination of the original variables, chosen such that the correlation between each pair $\left(U_{k},V_{k}\right)$ is maximized. The cross-covariance matrix between exposures and mediators $\Sigma_{EM}$, representing the linear relationship between the exposure and mediator variables, guides the optimization process to extract canonical variables that best preserve this relationship. In classical CCA, canonical components are orthogonal both within each dataset (components from the same dataset are uncorrelated) and between datasets for non-corresponding pairs (a component from one dataset is uncorrelated with non-matching components from the other dataset). In Sparse CCA, sparsity constraints relax these orthogonality conditions. As a result, different $U_{i}$ (or $V_{l}$) may be correlated, and cross-pair orthogonality, that is, $\mathrm{Cov}(U_{i},V_{l})=0\;\text{for}\;i\neq l,$ is not strictly enforced, but can be regarded as approximately orthogonal in practice, depending on the computational algorithm used (Rodosthenous *et al.* 2020). Additionally, by incorporating sparsity, SCCA allows for effective feature selection, which is essential in high-dimensional settings where many variables may be irrelevant or redundant.

Rather than using all possible canonical pairs for downstream mediation analysis, which may still be high-dimensional, we focus on the top *c* pairs with the strongest correlations. Here, *c* denotes the number of retained canonical pairs and is typically much smaller than min(p,q). In SCCA, sparsity is introduced through the penalty parameters $\lambda_{E}$ and $\lambda_{M}$, which impose $\ell_{1}$-type penalties on the loading matrices. These penalties shrink many coefficients to zero, thereby selecting only the most relevant variables. In our implementation, the estimation of the canonical variables (U,V) and the selection of the number of retained pairs *c* are performed sequentially, rather than jointly. Specifically, we consider a grid of penalty parameters $\left(\lambda_{E},\lambda_{M}\right)$ ranging from 0.1 to 0.9. To select the optimal combination, we embed the procedure within a 5-fold cross-validation framework (Yates *et al.* 2023). For each candidate pair $\left(\lambda_{E},\lambda_{M}\right)$, we perform the following steps:

- Step 1. Fit sparse canonical correlation analysis (SCCA) on the training data to obtain canonical directions and their corresponding correlations;
- Step 2. Determine the number of retained pairs *c* based on the cumulative explained squared canonical correlation criterion (e.g. 70%);
- Step 3. Construct the canonical variables $\left(U_{k},V_{k}\right)$ using the first *c* pairs;
- Step 4. Evaluate their predictive performance on the validation data;
- Step 5. Repeat Steps 1–4 across all folds and compute the average canonical correlation.

The optimal $\left(\lambda_{E},\lambda_{M}\right)$ is selected by maximizing the average canonical correlation. Finally, the optimal value *c* and the canonical components $\left(U_{k},V_{k}\right)$ for k=1,…,c are obtained by refitting the model on the full dataset using the chosen penalty parameters $\left(\lambda_{E},\lambda_{M}\right)$ (Härdle and Simar 2003, Wilms and Croux 2015).

The canonical data matrix for exposure can be written as


$$\begin{array}{c} U=\left[\begin{array}{cccc} U_{11} & U_{12} & \cdots & U_{1n}\\ U_{21} & U_{22} & \cdots & U_{2n}\\ \vdots & \vdots & \ddots & \vdots \\ U_{c1} & U_{c2} & \cdots & U_{cn} \end{array}\right]=W_{E}\cdot E, \end{array}$$

where $W_{E}$ is the sparse canonical loading or weight matrix with dimensions c×p, E is the data matrix of exposure variables with dimensions p×n, and U is the resulting matrix of canonical exposure variables with dimensions c×n, where the *k*th row $\left(U_{k1},U_{k2},\ldots ,U_{kn}\right)$ represents the canonical values of the *k*th canonical variable $U_{k}$.

Similarly, the canonical data matrix for mediator is


$$\begin{array}{c} V=\left[\begin{array}{cccc} V_{11} & V_{12} & \cdots & V_{1n}\\ V_{21} & V_{22} & \cdots & V_{2n}\\ \vdots & \vdots & \ddots & \vdots \\ V_{c1} & V_{c2} & \cdots & V_{cn} \end{array}\right]=W_{M}\cdot M, \end{array}$$

where $W_{M}$ is the sparse canonical loading or weight matrix with dimensions c×q, M is the data matrix of mediators with dimensions q×n, and V is the resulting matrix of canonical mediator variables with dimensions c×n, where the *l*th row $\left(V_{l1},V_{l2},\ldots ,V_{ln}\right)$ represents the canonical values of the *l*th canonical variable $V_{l}$.

### 2.2 Mediator and outcome models

After deriving the *c* pairs of canonical variables via SCCA, these variables are used to model the relationships between exposures, mediators, and the outcome in a structured manner. By transforming the original high-dimensional exposure and mediator variables into a lower-dimensional space, SCCA enhances interpretability while preserving key exposure-mediator associations. Let $U_{\text{var}}={\left(U_{1},\ldots ,U_{c}\right)}^{⊤}=W_{E}E_{\text{var}}$ and $V_{\text{var}}={\left(V_{1},\ldots ,V_{c}\right)}^{⊤}=W_{M}M_{\text{var}}$ denote the random vectors of the two sets of canonical variables. For each canonical mediator variable, we fit a linear regression model using the canonical exposure variables. That is:


$$\{\begin{array}{c} V_{1}=\alpha_{01}+\sum_{k=1}^{c}\alpha_{k1}U_{k}+\varepsilon_{1},\\ V_{2}=\alpha_{02}+\sum_{k=1}^{c}\alpha_{k2}U_{k}+\varepsilon_{2},\\ \;\vdots \\ V_{c}=\alpha_{0c}+\sum_{k=1}^{c}\alpha_{kc}U_{k}+\varepsilon_{c}. \end{array}.$$

Let $\alpha_{0}={\left(\alpha_{01},\ldots ,\alpha_{0c}\right)}^{⊤}$ be the vector of intercepts, $\varepsilon ={\left(\varepsilon_{1},\ldots ,\varepsilon_{c}\right)}^{⊤}$ be the vector of error terms, and let $\alpha =(\alpha_{ki})\in R^{c\times c}$ denote the regression coefficient matrix, where $\alpha_{ki}$ is the element in the *i*th row and *k*th column,

then the system of equations can be written as


(1) $$V_{\text{var}}=\alpha_{0}+\alpha U_{\text{var}}+\varepsilon_{M},$$

(2) $$Y=\beta_{0}+\gamma^{⊤}U_{\text{var}}+\delta^{⊤}V_{\text{var}}+\varepsilon_{Y},$$

where $\beta_{o}$ is the intercept, ε is the error term. Here, the direct effect is represented by the coefficient vector $\gamma ={\left(\gamma_{1},\ldots ,\gamma_{c}\right)}^{⊤}$, which quantifies the influence of the canonical exposure variables $U_{\text{var}}$ on the outcome *Y*. Similarly, the indirect effect, mediated through the canonical mediator variables $V_{\text{var}}$, is captured by the coefficient vector $\delta ={\left(\delta_{1},\ldots ,\delta_{c}\right)}^{⊤}$, which describes how variations in $V_{\text{var}}$ impact *Y*. This framework provides a clear distinction between direct and mediated effects, facilitating the analysis of complex causal relationships in high-dimensional mediation settings.

### 2.3 Estimation of direct and indirect effects

In linear mediation models, the indirect effect can be estimated in two equivalent ways. The difference-in-coefficients method calculates it as the reduction in the exposure’s effect on the outcome after adjusting for the mediator. In contrast, the product-of-coefficients method defines it as the product of the exposure’s effect on the mediator and the mediator’s effect on the outcome. Although both approaches yield the same point estimate, they differ in how standard errors are derived and in their flexibility. Simulation studies and theoretical work (Mackinnon *et al.* 2002) suggest that both perform similarly in large samples. However, the product-of-coefficients method has practical advantages: it extends more naturally to models with multiple mediators or interactions and works well with modern bootstrapping procedures, which provide more accurate confidence intervals, especially in small samples or when normality assumptions are violated. For these reasons, while both methods are valid, the product-of-coefficients approach has become the preferred method in contemporary research. In the following analysis, we adopt this approach to estimate indirect effects.

However, since $U_{\text{var}}$ and $V_{\text{var}}$ are derived canonical variables rather than the original exposure vector $E_{\text{var}}={\left(E_{1},\ldots ,E_{p}\right)}^{⊤}$ and mediator vector $M_{\text{var}}={\left(M_{1},\ldots ,M_{q}\right)}^{⊤}$, it is often necessary to map the estimated effects back to the original variables to ensure interpretability. This mapping step is crucial for identifying the direct effects of $E_{\text{var}}$ on *Y* and the indirect effects mediated through $M_{\text{var}}$.The following section provides a detailed explanation of this mapping process within the context of the original variables. We apply Bootstrap method to estimate the standard errors of the direct and indirect effects.

#### 2.3.1 Estimation of direct effects

The direct effect quantifies the portion of the influence of exposure $E_{i}$ on the outcome *Y* that is not mediated by $M_{j}$, meaning it represents the effect that occurs independently of the mediators. In the context of SCCA based mediation analysis, this effect is estimated using the canonical variables derived from the exposures.

Mathematically, the estimated outcome model (Eq. 2) is:


$$\hat{Y}=\hat{\beta_{0}}+{\hat{\gamma }}^{⊤}U_{\text{var}}+{\hat{\delta }}^{⊤}V_{\text{var}}.$$

Noting that $U_{\text{var}}=W_{E}E_{\text{var}},$ then we have


$$\hat{Y}=\hat{\beta_{0}}+{\hat{\gamma }}^{⊤}W_{E}E_{\text{var}}+{\hat{\delta }}^{⊤}V_{\text{var}}.$$

Therefore, the direct effect of $U_{\text{var}}$ is $\hat{\gamma }$, the direct effect of $E_{\text{var}}$ is $W_{E}^{⊤}\hat{\gamma }$, and the direct effect of $E_{i}$ on *Y* is


$${\hat{\text{Direct}\;\text{Effect}}}_{E_{i}}=\sum_{k=1}^{c}W_{E}^{\left(k,i\right)}{\hat{\gamma }}_{k},$$

where $W_{E}^{\left(i,k\right)}$, the elemant in the *i*th row and the *k*th column of $W_{E}$, is the weight indicating how much exposure $E_{i}$ contributes to the *k*th canonical variable $U_{k}$, and ${\hat{\gamma }}_{k}$ is the estimated coefficient from the outcome model representing the effect of $U_{k}$ on *Y*.

In this formulation, the canonical variables $U_{\text{var}}$ serve as lower-dimensional representations of the original exposure vector $E_{\text{var}}$, allowing for a more structured estimation of the direct effect. In practice, we select the number of canonical pairs *c* based on the cumulative explained squared canonical correlation, retaining the smallest number of pairs that achieve a prespecified threshold (e.g. 70%). To ensure stable estimation and avoid overfitting, we additionally require *c* to remain small relative to the sample size *n*. As a guideline, we recommend c≤n/10. The final choice of *c* is thus based on the correlation-based criterion, subject to this constraint. The direct effect of an individual exposure $E_{i}$ on *Y* is obtained by aggregating the weighted contributions of all canonical variables that capture variations in $E_{\text{var}}$. This approach mitigates multicollinearity and high dimensionality by focusing on the most relevant exposure–outcome relationships.

#### 2.3.2 Estimation of indirect effects

The indirect effect quantifies the portion of the relationship between exposure variables and the outcome that is mediated through mediators. Using the product-of-coefficient method, the indirect effect of $U_{\text{var}}$ through $V_{\text{var}}$ is ${\left({\hat{\delta }}^{⊤}\hat{\alpha }\right)}^{⊤}$. Because $U_{\text{var}}=W_{E}E_{\text{var}}$, the indirect effect of $E_{\text{var}}$ through $V_{\text{var}}$ is ${\left({\hat{\delta }}^{T}\hat{\alpha }W_{E}\right)}^{⊤}$. Then we try to find pathway of indirect effect of E via M on Y. To achieve this purpose, we consider a pseudo-mediation analysis using M as mediators, where the parameters of the mediation model will be estimated using Eq. (1) and the canonical relationship between V and M (Supplementary File 1, Appendix A):


$${\hat{\text{Indirect}\;\text{Effect}}}_{E_{i}\to M_{j}\to Y}=\sum_{k=1}^{c}\sum_{l=1}^{c}\delta_{l}\cdot m_{l,j}\cdot \alpha_{kl}\cdot W_{E}^{\left(i,k\right)},$$

where $m_{l,j}$ represent the element in the *l*th row and *j*th column of the matrix $W_{M}W_{M}^{+}$, quantifying how much the canonical variable $V_{l}$ influences the mediator $M_{j}$ after accounting for the normalization performed by the pseudo-inverse of $W_{M}$, that is, $W_{M}^{+}$. When the canonical variables, $V_{1},\ldots ,V_{c}$, are approximately orthogonal, the indirect effect can be simplified to $\sum_{k=1}^{c}\sum_{l=1}^{c}\delta_{l}\cdot \alpha_{kl}\cdot W_{E}^{\left(i,k\right)}$.

This formulation follows a multiplicative structure that reflects the sequential dependence among variables, linking the exposure $E_{i}$ to $U_{k}$, then $U_{k}$ to $V_{l}$, and finally, $V_{l}$ to *Y*. By incorporating the mapping relationships between canonical variables and the original variables, the estimated indirect effect can be translated back into the original mediation pathway, $E_{\text{var}}\to M_{\text{var}}\to Y$.

### 2.4 HDM-SCCA with screening (HDM-SCCA2)

To handle high-dimensional exposures and mediators, we propose a modified HDM-SCCA with a two-step feature screening procedure, referred to as HDM-SCCA2. (i) In the first step, an Elastic Net regression of the outcome Y on all exposures identifies a subset of exposures. (ii) In the second step, another Elastic Net regression of Y on the selected exposures and all mediators yields a subset of selected mediators. Finally, HDM-SCCA is applied to the selected exposures and mediators.

## 3 Simulation studies

We perform comprehensive simulation studies to evaluate the performance of the proposed HDM-SCCA method and the enhanced HDM-SCCA2 framework, and compare them with two benchmark approaches: the existing SPCA procedure (Sales 2017) and an oracle procedure that assumes knowledge of the true underlying models.

### 3.1 Design of simulation studies

We consider four scenarios, each involving *p* exposure variables and *q* mediator variables, among which $p_{1}$ exposures are truly associated with the outcome and $q_{1}$ mediators act as true mediators. The numbers of irrelevant exposures and mediators are denoted by $N_{e}=p-p_{1}$ and $N_{m}=q-q_{1}$, respectively. The sample size is either 500 or 1000, and 100 simulation replicates are performed. Here, we focus on Scenario 1 with the other scenarios discussed in Supplementary File 1. Scenario 1 represents a simplified setting in which the coefficients $\beta_{E_{i}}$, $\beta_{M_{j}}$, and α are constant across all relevant exposures $\left(E_{i}\in E_{\text{var}}\right)$ and mediators $\left(M_{j}\in M_{\text{var}}\right)$. In this scenario, all relevant exposures and mediators involved in mediation pathways are associated with the outcome *Y*.

### 3.2 Generation of exposure

The exposure variables consist of both relevant and irrelevant components. Relevant exposures associated with the outcome are generated from a multivariate normal distribution with mean 0, variance 1, and a structured covariance matrix with pairwise correlation 0.5. Irrelevant exposures are generated independently from a standard normal distribution with an identity covariance matrix.

### 3.3 Generation of mediators

The mediator variables are generated as linear combinations of the exposure variables. Each relevant mediator $M_{j}$ is associated with a subset of exposures, with additive noise. The resulting matrix is standardized to ensure consistency across variables. To be specific, for $j=1,\ldots ,q_{1},$


$$\begin{array}{c} M_{j}=\sum_{i=j}^{j+l}\alpha_{i,j}E_{i}+\epsilon_{M_{j}},\\ \epsilon_{M_{j}}∼N\left(0,\sigma_{M}^{2}\right),\;\sigma_{M}^{2}=\{\begin{array}{cc} 0.5 & \text{Simulation}\;\text{Scenario}\;1,\\ 1 & \text{Simulation}\;\text{Scenario}\;2. \end{array} \end{array}$$

where *l* is defined as 1 for simulation Scenario 1 and 2 for simulation Scenario 2.

The correlation among mediators arises from both structured exposure effects and inherent noise dependencies. In Scenario 1, each mediator $M_{j}$ is influenced by $E_{j}$ and $E_{j+1}$ via the coefficient matrix α, inducing correlations through shared exposures. In Scenario 2, each mediator $M_{j}$ is influenced by $E_{j}$, $E_{j+1}$, and $E_{j+2}$, also inducing correlations via shared exposures. Additionally, the error term $\epsilon_{M}$ follows a multivariate normal distribution with covariance $\Sigma_{M}$, where off-diagonal elements (0.3) introduce moderate dependencies. Consequently, $M_{\text{var}}$ exhibits a structured correlation pattern shaped by both exposure driven effects and stochastic influences.

### 3.4 Generation of the outcome variable

The outcome *Y* is generated as a linear combination of $p_{1}$ relevant exposures and $q_{1}$ relevant mediators with fixed coefficients, plus Gaussian noise to account for random variability:


$$\begin{array}{c} Y=\sum_{j=1}^{p_{1}}\beta_{E_{j}}E_{j}+\sum_{k=1}^{q_{1}}\beta_{M_{k}}M_{k}+\epsilon_{Y},\\ \epsilon_{Y}∼N\left(0,\sigma_{Y}^{2}\right),\;\sigma_{Y}^{2}=\{\begin{array}{cc} 0.2^{2} & \text{Simulation}\;\text{Scenario}\;1,\\ 1 & \text{Simulation}\;\text{Scenario}\;2. \end{array} \end{array}$$

### 3.5 Metrics for comparison

The oracle estimates, based on the true exposure and mediator variables, provide a gold standard for comparison, representing the best achievable estimates in our simulation setting. Direct effects are calculated from the regression analysis of *Y* on the true exposure variables ($E_{\text{true}}$) and true mediator variables ($M_{\text{true}}$). The indirect effects are calculated as the product of the coefficients from the regression of $M_{\text{true}}$ on $E_{\text{true}}$ and the coefficients of *Y* on $E_{\text{true}}$ and $M_{\text{true}}$. The performance of different methods is evaluated using the True Positive Rate (TPR) and False Positive Rate (FPR) for selected variables, and by quantifying bias in the estimated direct and indirect effects for exposures and mediators truly associated with the outcome.

## 4 Simulation results

For simulation Scenario 1, we fix $p_{1}=6$ true exposures and $q_{1}=5$ true mediators. The numbers of irrelevant exposures $N_{e}$ and irrelevant mediators $N_{m}$ vary across three configurations: 10, 50, and 1000. The coefficients of the relevant exposures on the outcome are set to $\beta_{E_{j}}=0.15$, while the coefficients of the relevant mediators are set to $\beta_{M_{k}}=0.2$. Each relevant mediator $M_{j}$ depends on $E_{j}$ and $E_{j+1}$ through the coefficient matrix α, inducing correlation among mediators via shared exposures. All nonzero elements of α are set to 0.5.

The performance of HDM-SCCA, HDM-SCCA2, SPCA, and the oracle method in estimating direct and indirect effects for sample size is compared in Table 1 for n=500 and Supplementary File 1 (Table S1) for n=1000. The values in parentheses below the bias estimates represent bootstrap standard deviations, reflecting estimation variability. All methods perform similarly well in estimating direct effects, with small bias across noise levels. We note, however, that HDM-SCCA2 can exhibit slightly larger bias or variability in estimating direct effects compared to HDM-SCCA, and in some cases performs comparably to or worse than SPCA. However, for indirect effects, HDM-SCCA and HDM-SCCA2 substantially outperform SPCA. In particular, SPCA consistently overestimates indirect effects and exhibits larger standard errors, indicating reduced precision. These results suggest that the SCCA-based frameworks are more effective at capturing mediation pathways between exposures and the outcome.

**Table 1:** Comparisons of bias and standard deviation (in parentheses) for different methods in estimating direct and indirect effects under Scenario 1 (sample size n=500).

		$N_{e}=10,N_{m}=10$			$N_{e}=50,N_{m}=50$			$N_{e}=1000,N_{m}=1000$		
	Oracle	HDM-SCCA2	HDM-SCCA	SPCA	HDM-SCCA2	HDM-SCCA	SPCA	HDM-SCCA2	HDM-SCCA	SPCA
Direct effects										
E1	0.018 (0.013)	−0.030 (0.031)	−0.004 (0.017)	0.001 (0.006)	−0.026 (0.027)	−0.000 (0.012)	0.008 (0.006)	−0.007 (0.019)	−0.002 (0.010)	0.010 (0.005)
E2	−0.013 (0.014)	0.031 (0.031)	0.011 (0.017)	0.001 (0.006)	0.029 (0.027)	0.012 (0.012)	0.008 (0.006)	0.011 (0.019)	0.001 (0.010)	0.010 (0.005)
E3	−0.001 (0.015)	0.004 (0.031)	0.002 (0.017)	0.002 (0.006)	−0.010 (0.027)	0.011 (0.012)	0.008 (0.006)	0.002 (0.019)	0.002 (0.010)	0.011 (0.005)
E4	0.017 (0.015)	−0.002 (0.031)	0.005 (0.017)	0.001 (0.006)	−0.004 (0.027)	0.008 (0.012)	0.008 (0.006)	−0.003 (0.019)	0.002 (0.010)	0.012 (0.005)
E5	−0.014 (0.015)	0.033 (0.031)	0.009 (0.017)	0.002 (0.006)	0.025 (0.027)	0.009 (0.012)	0.008 (0.006)	0.009 (0.019)	0.001 (0.010)	0.010 (0.005)
E6	−0.008 (0.013)	−0.026 (0.031)	−0.013 (0.017)	0.001 (0.006)	−0.017 (0.027)	−0.003 (0.012)	0.008 (0.006)	−0.011 (0.019)	−0.001 (0.010)	0.009 (0.005)
Indirect effects										
E1 M1	−0.013 (0.016)	−0.022 (0.051)	−0.031 (0.048)	0.452 (0.164)	−0.039 (0.048)	−0.005 (0.046)	0.022 (0.163)	−0.039 (0.036)	−0.031 (0.040)	0.011 (0.164)
E2 M1	−0.026 (0.016)	0.029 (0.051)	0.005 (0.048)	0.455 (0.164)	0.009 (0.048)	0.009 (0.046)	0.021 (0.163)	0.002 (0.036)	−0.021 (0.040)	0.010 (0.164)
E2 M2	−0.007 (0.015)	0.025 (0.051)	0.018 (0.048)	0.155 (0.164)	0.033 (0.048)	0.012 (0.046)	0.359 (0.163)	−0.011 (0.036)	−0.021 (0.040)	0.363 (0.164)
E3 M2	−0.022 (0.015)	0.018 (0.051)	0.012 (0.048)	0.156 (0.164)	0.004 (0.048)	0.008 (0.046)	0.359 (0.163)	−0.023 (0.036)	−0.019 (0.040)	0.369 (0.164)
E3 M3	0.000 (0.016)	0.009 (0.051)	0.001 (0.048)	0.003 (0.164)	0.001 (0.048)	0.005 (0.046)	0.016 (0.163)	−0.014 (0.036)	−0.018 (0.040)	0.015 (0.164)
E4 M3	−0.020 (0.016)	0.017 (0.051)	−0.017 (0.048)	0.004 (0.164)	0.013 (0.048)	0.009 (0.046)	0.016 (0.163)	−0.011 (0.036)	−0.017 (0.040)	0.016 (0.164)
E4 M4	−0.026 (0.016)	0.026 (0.051)	0.021 (0.048)	−0.004 (0.164)	0.025 (0.048)	0.011 (0.046)	0.016 (0.163)	−0.011 (0.036)	−0.017 (0.040)	0.030 (0.164)
E5 M4	−0.026 (0.016)	0.021 (0.051)	0.051 (0.048)	−0.003 (0.164)	0.039 (0.048)	0.004 (0.046)	0.015 (0.163)	−0.004 (0.036)	−0.017 (0.040)	0.029 (0.164)
E5 M5	−0.017 (0.015)	0.024 (0.051)	−0.019 (0.048)	0.003 (0.164)	0.011 (0.048)	0.005 (0.046)	0.006 (0.163)	0.008 (0.036)	−0.020 (0.040)	0.011 (0.165)
E6 M5	−0.000 (0.015)	−0.019 (0.051)	−0.039 (0.048)	0.001 (0.164)	−0.032 (0.048)	−0.003 (0.046)	0.006 (0.163)	−0.031 (0.036)	−0.034 (0.040)	0.011 (0.165)

This performance difference reflects fundamental methodological distinctions. In this simulation, mediators are generated as linear combinations of exposures with added Gaussian noise. SCCA explicitly maximizes the correlation between exposures and mediators while enforcing sparsity, enabling it to identify mediators that carry indirect effects even in high-noise settings. In contrast, SPCA prioritizes variance explained within the mediator block and does not explicitly account for exposure–mediator relationships, leading to systematic bias in indirect effect estimation when many irrelevant variables are present. The additional screening step in HDM-SCCA2 further introduces a trade-off: while the Elastic Net pre-screening effectively removes irrelevant variables and improves selection accuracy, it may also discard variables that are weak individually but jointly informative. As a result, some multivariate structure is lost prior to SCCA, which can negatively affect the estimation of direct effects. In contrast, HDM-SCCA operates on the full set of variables and better preserves joint dependence, leading to more stable direct effect estimates.

We also evaluate the True positive rate (TPR) and false positive rate (FPR) for identifying exposures and mediators using the proposed methods (Supplementary File 1, Tables S2 and S3). Specifically, for each simulation replicate, we identify the selected exposures and mediators from the SCCA model and compare them with the corresponding true sets defined by the data-generating mechanism. TPR and FPR are then computed at the level of the original variables and averaged across replicates. HDM-SCCA achieves high TPRs ranging from 0.97 to 1.00 and low FPRs between 0.00 and 0.08 across all configurations. HDM-SCCA2 further improves performance, achieving a TPR of 1 and an FPR of 0 for both exposures and mediators in all settings, demonstrating the effectiveness of the two-step Elastic Net screening procedure.

To assess robustness under more complex conditions, three additional scenarios are considered and reported in Supplementary File 1  (Appendix B). Scenario 2 allows coefficients to vary across exposures and mediators and includes relevant variables that are not associated with *Y*. In Scenario 3, some relevant exposures are not associated with *Y*, while all relevant mediators remain associated with *Y*. In contrast, in Scenario 4, some relevant mediators are not associated with *Y*, but all relevant exposures are associated with *Y*. Detailed results for these scenarios are provided in Supplementary File 1 (Tables S4–S9). We further examine the sensitivity of the method to the choice of canonical pair threshold and to heterogeneous mediator error variances in Supplementary File 1  (Appendix C). These additional analyses demonstrate that the proposed approach remains stable under a range of threshold choices and variance structures.

## 5 Real data

Alcohol-associated liver disease (ALD) is a leading cause of liver-related mortality and liver transplantation worldwide. The severity of ALD spans a broad spectrum, ranging from hepatic steatosis to steatohepatitis, progressive fibrosis, and ultimately cirrhosis. ALD is influenced by multiple established risk factors, including the extent of alcohol consumption, sex, age, obesity, type 2 diabetes mellitus, gut microbial dysbiosis, and genetic variants (Huang *et al.* 2023). Beyond these factors, growing evidence suggests that environmental pollutants also play an important role in ALD pathogenesis, as many pollutants act as metabolic and endocrine disruptors and contribute to liver injury and metabolic dysfunction (Beier *et al.* 2025).

In this study, we investigated the disease-modifying effects of environmental pollutants on ALD severity and elucidated potential underlying mechanisms by integrating untargeted exposome profiling with metabolomic analysis. Specifically, we aimed to investigate both the direct effects of environmental chemical exposures and their indirect effects mediated through circulating metabolites on ALD severity. Defeat Alcoholic Steatohepatitis (DASH) is a multicenter, randomized, double-blind controlled trial (Dasarathy *et al.* 2020). Previously collected and de-identified plasma samples from 114 DASH participants with moderate to severe ALD were used for this cross-sectional analysis. Demographic and clinical characteristics of the study population are summarized in Supplementary File 1 (Table S11). The Model for End-Stage Liver Disease (MELD) score was used as the primary outcome, as it is a well-established and widely accepted measure of liver disease severity. The metabolomic dataset comprised 9894 distinct metabolites considered as potential mediators, while the exposome dataset included 685 environmental chemicals serving as exposure variables (Supplementary File 2). The high-dimensional nature of the dataset presents a challenge in identifying relevant mediators and exposures that influence the MELD score while effectively accounting for the substantial noise inherent in large-scale omics data (Vatsalya *et al.* 2020).

To address the high dimensionality, we applied a two-step Elastic Net screening procedure, as in HDM-SCCA2, to identify exposures and mediators associated with the MELD score. Elastic Net with α=0.5 selected 118 exposures and 121 mediators. All variables were standardized, and the MELD score was logit-transformed to reduce skewness.

We applied SCCA to investigate associations between chemical exposures and metabolites in relation to the MELD score. To account for variability in model tuning and selection, the entire analysis pipeline was repeated 50 times using bootstrap samples. In each run, tuning parameters, including the exposure and mediator penalties ($\lambda_{E}$, $\lambda_{M}$) and the number of canonical pairs (*c*), were selected via cross-validation. Across runs, the resulting canonical correlations were consistently high, suggesting a stable underlying association structure between the exposure and mediator sets. For interpretability, we thresholded canonical weights within each run by setting coefficients with absolute values below 0.1 to zero, yielding a reduced subset of chemicals and metabolites for subsequent analysis. Indirect effects were then estimated using the product-of-coefficients approach, and their uncertainty was quantified using bootstrap-based standard errors. Statistical significance was assessed using Wald-type tests based on these bootstrap standard errors, with two-sided *P*value obtained under a normal approximation. To account for multiple comparisons across exposure–mediator pairs, *P*value were further adjusted using the Benjamini–Hochberg procedure. The total computational time for the 50 repeated runs was approximately 530 seconds, indicating that the proposed procedure is computationally manageable for datasets of this scale.

Across the 50 repeated runs, no chemical exhibited a statistically significant direct effect on the MELD score, suggesting limited evidence for a direct association after accounting for mediator pathways and model selection variability. Several mediation pathways show statistically significant indirect effects and moderate selection stability with selection rates greater than 0.4 (Table 2). We conducted an $R^{2}$-based sensitivity analyses to evaluate the robustness of these pathways to potential unmeasured confounding (Cinelli and Hazlett 2020, Cinelli *et al.* 2024). As shown in Table 3, most pathways are characterized by moderate exposure–mediator associations ($R_{a}^{2}$) but weak mediator–outcome associations ($R_{b}^{2}$), leading to low robustness values ($RV_{\mathrm{path}}$). In contrast, pathway involving acetic acid exhibits stronger mediator–outcome associations and substantially higher robustness values, suggesting that this effect is less sensitive to unmeasured confounding and therefore more reliable.

**Table 2:** Indirect effects of selected chemical exposures through metabolites.

Exposure	Mediator	Indirect Effect	SE	Adjusted-*P*
Anthraquinone	Dihydrobiopterin	0.35	0.07	5.53E−3
Tris(chloropropyl) phosphate (TCPP)	(Iso)Vanillic acid	0.40	0.07	3.99E−3
Anthraquinone	Acetic acid	0.36	0.06	7.08E−3
Tris(chloropropyl) phosphate (TCPP)	Polyphenol tannin	0.36	0.08	9.73E−3
Tris(chloropropyl) phosphate (TCPP)	5-Ethynyl-5–(1-propynyl)-2,2’-bithiophene	0.38	0.08	4.38E−3
Diallate_trans	Dihydrobiopterin	0.29	0.06	4.61E−3
Tris(chloropropyl) phosphate (TCPP)	12-Cyclohexanedione	0.35	0.07	5.41E−3
1,4-Naphthoquinone	Dihydrobiopterin	0.30	0.06	7.66E−3

**Table 3:** Sensitivity analysis of mediation pathways.

Exposure	Mediator	$R_{a}^{2}$	$R_{b}^{2}$	RV${\;}_{a}$	RV${\;}_{b}$	RV${\;}_{\text{path}}$
Anthraquinone	Dihydrobiopterin	0.1509	0.0107	0.3419	0.0989	0.0989
Tris(chloropropyl) phosphate (TCPP)	(Iso)Vanillic acid	0.2012	0.0070	0.3915	0.0807	0.0807
Anthraquinone	Acetic acid	0.0380	0.0566	0.1801	0.2168	0.1801
Tris(chloropropyl) phosphate (TCPP)	Polyphenol tannin	0.0642	0.0067	0.2299	0.0791	0.0791
Tris(chloropropyl) phosphate (TCPP)	5-Ethynyl-5–(1-propynyl)-2,2’-bithiophene	0.0776	0.0208	0.2511	0.1357	0.1357
Diallate_trans	Dihydrobiopterin	0.1357	0.0066	0.3726	0.0786	0.0786
Tris(chloropropyl) phosphate (TCPP)	12-Cyclohexanedione	0.0509	0.0053	0.2065	0.0709	0.0709
1,4-Naphthoquinone	Dihydrobiopterin	0.1558	0.0049	0.3472	0.0678	0.0678

This interpretation is consistent with prior work suggesting that acetic acid (acetate at physiological pH) may contribute to liver injury through several mechanisms. Acetate is a product of the oxidative metabolism of alcohol, and its formation has been linked to many of the pathological changes seen in the livers of individuals chronically consuming alcohol (Moghe *et al.* 2011). Acetate can be further metabolized to acetyl-CoA, a cofactor required for lipid biosynthesis and histone acetylation of inflammatory genes, providing a mechanistic link between acetate production, steatosis and inflammation in the liver (Kendrick *et al.* 2010, Wang *et al.* 2025, 2026). Identification of acetate as a mediator of anthraquinone on MELD score suggests that exposure to this environmental toxicant may increase the rate of acetate formation or decrease the rate of acetate elimination. Thus, anthraquinone may alter metabolic processes that increase acetate or related intermediates, which in turn contribute to liver damage.

## 6 Conclusions and discussions

This study addresses the challenges of mediation analysis with high dimensional exposures and mediators by proposing a new SCCA-based mediation analysis, HDM-SCCA. An extended version, HDM-SCCA2, further improves the identification of relevant exposures and mediators by incorporating a two-step feature screening procedure. Through simulation studies with various sample sizes and noise levels, the HDM-SCCA approach demonstrated superior accuracy, robustness, and interpretability compared to existing methods such as SPCA. By capturing both direct and mediated effects in a high-dimensional setting, this framework provides an interpretable approach for understanding exposure-mediator-outcome relationships.

This framework has broad interdisciplinary potential. It can study complex diseases in biomedicine, analyse pollutant effects in environmental science, and model behavioral or economic mediators in social sciences and economics. By extending high-dimensional mediation analysis to diverse domains, it can improve decision-making, predictive modeling, and causal inference across public health, environmental policy, and social and economic systems.

While the current application is cross-sectional, future work could refine this approach by integrating dynamic modeling to capture complex high-dimensional relationships. Extending the methodology to longitudinal studies would allow the assessment of temporal or causal relationships, including dynamic exposure–mediator–outcome interactions (Mittinty and Vansteelandt 2020, Den Teuling *et al.* 2023). Incorporating time-varying effects and latent variable modeling could further enhance its ability to track changes over time and across multiple mediators (Di Maria and Didelez 2024).

However, HDM-SCCA has some limitations. One key assumption is that it relies on linear relationships between the variables. In many applications, these linear approximations are able to capture the primary signal of interest, especially when the goal is to identify major mediation pathways. Nevertheless, we acknowledge that the proposed approach may have limited sensitivity to nonlinear relationships, and as a result, certain complex mediation effects could be missed. Incorporating nonlinear structures, such as kernel-based extensions of CCA or nonlinear mediation models, represents a promising direction for future work.

## Supplementary Material

btag474_Supplementary_Data
