# Supplementary material for: Sparse CCA-based mediation analysis with high-dimensional exposures and mediators
Source: Bioinformatics. 2026 Jun 30;42(7):btag474. doi: 10.1093/bioinformatics/btag474 (PMC13384060; doi:10.1093/bioinformatics/btag474)
Supplement: btag474_Supplementary_Data [file btag474_supplementary_data.zip › SupplementaryFile1.pdf]

Supplementary File 1: Sparse CCA-Based Mediation Analysis with High-Dimensional Exposures and Mediators

Appendix A Detailed Steps of Indirect Effect Estimation

To estimate the indirect effects at level of the original variables, we consider the following mediation analysis. The mediators model using $\mathbf{M}_{\text{var}}$ as mediators and $\mathbf{U}_{\text{var}}$ as exposures and the outcome model are

$$\begin{cases} \mathbf{M}_{\text{var}} = \eta_0 + \eta \mathbf{U}_{\text{var}} + \epsilon_M, \\ Y = \beta_0 + \gamma^\top \mathbf{U}_{\text{var}} + \Delta^\top \mathbf{M}_{\text{var}} + \epsilon_Y. \end{cases} \quad \text{(Eq. S1)}$$

As there may be more mediators than the number of observations, we are not able to estimate the parameters in Eq. S1 directly. Instead, we estimate these parameters using the estimates in Eq.1 of the main text and the canonical relationship between $\mathbf{V}_{\text{var}}$ and $\mathbf{M}_{\text{var}}$. That is,

$$\begin{cases} \widehat{\mathbf{V}}_{\text{var}} = \hat{\alpha_0} + \hat{\boldsymbol{\alpha}} \mathbf{U}_{\text{var}}, \\ \mathbf{V}_{\text{var}} = W_M \cdot \mathbf{M}_{\text{var}}. \end{cases}$$

This set of equations connects the mediator $\mathbf{M}_{\text{var}}$ to $\mathbf{U}_{\text{var}}$ through $\mathbf{V}_{\text{var}}$. To get the estimated function of $\mathbf{M}_{\text{var}}$, we use $\hat{\alpha_0}$ and $\hat{\boldsymbol{\alpha}}$ which are estimated from Eq.1, setting

$$W_M \cdot \widehat{\mathbf{M}}_{\text{var}} = \hat{\alpha_0} + \hat{\alpha} \mathbf{U}_{\text{var}}.$$

Using the minimum norm solution of the Moore-Penrose pseudoinverse, we have

$$\widehat{\mathbf{M}}_{\text{var}} = W_M^+ \hat{\alpha_0} + W_M^+ \hat{\alpha} \mathbf{U}_{\text{var}}, \quad \text{(Eq. S2)}$$

where W_M^+ is the Moore-Penrose pseudoinverse of W_M, which is $W_M^\top (W_M W_M^\top)^{-1}$.

The outcome model, using $\mathbf{M}_{\text{var}}$ as a mediator, is modeled by:

$$Y = \beta_0 + \gamma^T \mathbf{U}_{\text{var}} + \Delta^T \mathbf{M}_{\text{var}} + \epsilon_Y. \quad \text{(Eq. S3)}$$

Then the indirect effect of $\mathbf{U}_{\text{var}}$ through $\mathbf{M}_{\text{var}}$ is $(\hat{\Delta}^T W_M^+ \hat{\alpha})^\top$. Now we need to find the estimate $\hat{\Delta}^T$. Requiring the indirect effects should be the same as the indirect effect of $\mathbf{U}_{\text{var}}$ derived by using $\mathbf{U}_{\text{var}}$ and $\mathbf{V}_{\text{var}}$ in the mediator model and outcome model, which is $(\hat{\delta}^T \hat{\alpha})^\top$, thus, we have the relationship: $\hat{\Delta}^\top W_M^+ \hat{\alpha} = \hat{\delta}^\top \hat{\alpha}$, which implies $\hat{\Delta}^\top W_M^+ = \hat{\delta}^\top$. Equivalently, $(W_M^+)^\top \hat{\Delta} = \hat{\delta}$. Using the minimum-norm solution and the facts of pseudoinverse, i.e. $(W^+)^+ = W$, and $(W^+)^\top = (W^\top)^+$, we have $\hat{\Delta} = W_M^\top \hat{\delta}$. Therefore, the indirect effect of $\mathbf{U}_{\text{var}}$ through M is $(\hat{\Delta}^\top W_M^+ \hat{\alpha})^\top = (\hat{\delta}^\top W_M W_M^+ \hat{\alpha})^\top$, and the indirect effect of $\mathbf{E}_{\text{var}}$ through M is $(\hat{\delta}^\top W_M W_M^+ \hat{\alpha} W_E)^\top$. When the rows of W_M are orthogonal, this can be further simplified as $(\hat{\delta}^\top \hat{\alpha} W_E)^\top$.

Appendix B Supplementary Simulation Scenerios

B.1 Simulation Scenario 1, sample size $n = 1000$, $p_1 = 6$, $q_1 = 5$, $p = N_e + p_1$, $q = N_m + q_1$

For the larger sample size $n = 1000$, Table S1 indicates that HDM-SCCA, HDM-SCCA2, and SPCA exhibit comparable performance in estimating direct effects, with consistently small bias across all noise levels. In contrast, HDM-SCCA and HDM-SCCA2 achieve substantially improved performance in estimating indirect effects, characterized by smaller bias and reduced variability. SPCA continues to overestimate indirect effects and demonstrates larger standard errors, reflecting lower estimation precision. In terms of variable selection, HDM-SCCA (Table S2) attains high true positive rates for both exposures and mediators, ranging from 0.97 to 1.00, with low false positive rates across all configurations. HDM-SCCA2 (Table S3) further improves identification accuracy, achieving a true positive rate of 1 and a false positive rate of 0 for both exposures and mediators, highlighting the effectiveness of the two-step Elastic Net screening procedure in high-dimensional settings with larger sample sizes.

B.2 Simulation Scenario 2

We then conducted a simulation study to test whether HDM-SCCA2 with two step elastic net screening can still accurately estimate the effects with complex settings. The configuration was set with $p_1 = 5$ and $p_2 = 3$. Instead of fixing the coefficients, we allowed the coefficients of exposures on the outcome to vary for each exposure, and

Table S1: Comparisons of bias and standard deviation (in parentheses) for different methods in estimating the direct and indirect effects under Scenario 1 (sample size $n = 1000, p = N_e + 6, q = N_m + 5$)

		$N_e = 10, N_m = 10$			$N_e = 50, N_m = 50$			$N_e = 1000, N_m = 1000$		
	Oracle	HDM-SCCA2	HDM-SCCA	SPCA	HDM-SCCA2	HDM-SCCA	SPCA	HDM-SCCA2	HDM-SCCA	SPCA
**Direct Effects**										
E1	-0.011 (0.010)	-0.033 (0.033)	-0.019 (0.026)	0.001 (0.004)	-0.025 (0.026)	-0.000 (0.012)	0.008 (0.004)	-0.036 (0.040)	-0.003 (0.019)	0.013 (0.004)
E2	-0.007 (0.012)	0.038 (0.033)	0.019 (0.026)	0.001 (0.004)	0.027 (0.026)	0.014 (0.012)	0.008 (0.004)	0.034 (0.040)	0.003 (0.019)	0.010 (0.004)
E3	0.011 (0.012)	0.001 (0.033)	0.007 (0.026)	0.001 (0.004)	-0.003 (0.026)	0.011 (0.012)	0.007 (0.004)	0.005 (0.040)	0.003 (0.019)	0.010 (0.004)
E4	0.010 (0.012)	0.002 (0.033)	0.008 (0.026)	0.001 (0.004)	-0.001 (0.026)	0.009 (0.012)	0.007 (0.004)	-0.002 (0.040)	0.002 (0.019)	0.011 (0.004)
E5	0.008 (0.011)	0.034 (0.033)	0.021 (0.026)	0.001 (0.004)	0.026 (0.026)	0.011 (0.012)	0.008 (0.004)	0.037 (0.040)	0.004 (0.019)	0.010 (0.004)
E6	0.005 (0.011)	-0.034 (0.033)	-0.023 (0.026)	0.000 (0.004)	-0.028 (0.026)	-0.001 (0.012)	0.007 (0.004)	-0.029 (0.040)	-0.004 (0.019)	0.009 (0.004)
**Indirect Effects**										
E1 M1	-0.004 (0.012)	-0.023 (0.052)	-0.008 (0.047)	0.457 (0.166)	-0.026 (0.047)	-0.001 (0.049)	0.026 (0.166)	-0.025 (0.058)	-0.013 (0.094)	0.022 (0.166)
E2 M1	-0.008 (0.012)	0.031 (0.052)	-0.004 (0.047)	0.459 (0.166)	0.031 (0.047)	0.016 (0.049)	0.026 (0.166)	0.031 (0.058)	0.015 (0.094)	0.019 (0.166)
E2 M2	-0.021 (0.012)	0.025 (0.052)	0.014 (0.047)	0.161 (0.167)	0.030 (0.047)	0.016 (0.049)	0.362 (0.166)	0.029 (0.058)	0.016 (0.094)	0.368 (0.166)
E3 M2	-0.021 (0.012)	0.026 (0.052)	0.008 (0.047)	0.161 (0.167)	0.006 (0.047)	0.007 (0.049)	0.360 (0.166)	0.025 (0.058)	0.017 (0.094)	0.369 (0.166)
E3 M3	-0.008 (0.010)	0.012 (0.052)	0.000 (0.047)	-0.013 (0.167)	-0.012 (0.047)	0.007 (0.049)	0.012 (0.165)	0.011 (0.058)	0.017 (0.094)	0.010 (0.166)
E4 M3	-0.002 (0.010)	0.021 (0.052)	0.004 (0.047)	-0.013 (0.167)	0.003 (0.047)	0.008 (0.049)	0.012 (0.165)	-0.025 (0.058)	0.016 (0.094)	0.011 (0.166)
E4 M4	-0.018 (0.011)	0.031 (0.052)	0.011 (0.047)	0.005 (0.166)	0.011 (0.047)	0.015 (0.049)	0.009 (0.165)	0.036 (0.058)	0.016 (0.094)	0.030 (0.166)
E5 M4	-0.013 (0.011)	0.026 (0.052)	0.016 (0.047)	0.005 (0.166)	0.025 (0.047)	0.018 (0.049)	0.009 (0.165)	0.022 (0.058)	0.019 (0.094)	0.030 (0.166)
E5 M5	-0.006 (0.012)	0.029 (0.052)	-0.001 (0.047)	0.007 (0.166)	0.016 (0.047)	0.017 (0.049)	0.013 (0.165)	0.026 (0.058)	0.019 (0.094)	0.010 (0.166)
E6 M5	-0.010 (0.012)	-0.021 (0.052)	-0.008 (0.047)	0.007 (0.166)	-0.031 (0.047)	-0.001 (0.049)	0.013 (0.165)	-0.018 (0.058)	-0.013 (0.094)	0.009 (0.166)

similarly, the coefficients of mediators on outcome to differ for each mediator. The coefficients for exposures on outcome were set as $(0.15, 0.18, 0.25, 0.22, 0)$, and the coefficients for mediators on outcome were $(0, 0.3, 0.45)$, which indicates E_5 and M_1 do not correlate with Y. The coefficients for E_i on M_i were set to 0.2, E_{i+1} on M_i to 0.15, and E_{i+2} on M_i to 0.4.

We also aimed to test whether HDM-SCCA2 could accurately capture the relationships when some exposures and mediators correlate with each other but do not have a relationship with Y. In this scenario, we set E_5 to correlate with M_3, but not with Y, and M_1 to correlate with E_1, E_2, E_3, but not with Y.

Table S2: TPR and FPR for selected exposures and mediators using HDM-SCCA under simulation Scenario 1

n	N_e	N_m	TPR		FPR	
			Exposures	Mediators	Exposures	Mediators
500	10	10	1.00	1.00	0.00	0.00
	50	50	1.00	0.97	0.08	0.01
	1000	1000	0.99	1.00	0.00	0.00
1000	10	10	1.00	1.00	0.00	0.00
	50	50	1.00	0.97	0.01	0.01
	1000	1000	1.00	1.00	0.00	0.00

Table S3: TPR and FPR for selected exposures and mediators using HDM-SCCA2 under simulation Scenario 1

n	N_e	N_m	TPR		FPR	
			Exposures	Mediators	Exposures	Mediators
500	10	10	1.00	1.00	0.00	0.00
	50	50	1.00	1.00	0.00	0.00
	1000	1000	1.00	1.00	0.00	0.00
1000	10	10	1.00	1.00	0.00	0.00
	50	50	1.00	1.00	0.00	0.00
	1000	1000	1.00	1.00	0.00	0.00

$$M_j = \sum_{i=j}^{j+2} \alpha_{i,j} E_i + \epsilon_{M_j}, \quad \epsilon_{M_j} \sim \mathcal{N}(0,1), \quad j = 1,2,3$$

$$Y = \sum_{j=1}^{p_1} \beta_{E_j} E_j + \sum_{k=1}^{p_2} \beta_{M_k} M_k + \epsilon_Y, \quad \epsilon_Y \sim \mathcal{N}(0,1).$$

Across various scenarios with different levels of irrelevant variables, the two-step Elastic Net screening method followed by HDM-SCCA provides estimates for both direct and indirect effects that are almost as accurate as those from the oracle model (Tables S4 and S5). This holds true even when the number of irrelevant variables reaches 500. The TPR for both exposures and mediators remains consistently high, close to 1 across all noise levels, demonstrating the method's strong ability to identify truly relevant variables. Additionally, the FPR for both exposures and mediators remains relatively low, indicating that the method effectively avoids falsely selecting irrelevant variables. This combination of high TPR and low FPR suggests that the two-step Elastic Net plus HDM-SCCA method is highly reliable and robust, even in the presence of a large number of irrelevant variables.

B.3 Simulation Scenario 3

The configuration was set with $p_1 = 5$ and $q_1 = 3$. In this simulation, one exposure (E_4) is set to correlate with mediators (M_1, M_2, and M_3) but is not correlated with the outcome variable (Y). All other exposures and mediators, however, are correlated with each other and with Y. Specifically, the coefficients for the exposures on Y are set as $(0.15, 0.32, 0.28, 0, 0.2)$, where E_4 has no direct effect on Y. For the mediators, the coefficients on Y are $(0.7, 0.8, 0.5)$, with each mediator having varying degrees of influence on the outcome. The relationships between the exposures and the mediators are modeled as follows: the coefficient for E_i on M_i is set to 0.5, for E_i on M_{i+1} to 0.6, and for E_i on M_{i+2} to 0.4. This design allows for testing the ability of the method to accurately capture the relationships in a scenario where one exposure does not directly correlate with the outcome, but all mediators do. The results showing the bias of the estimates for both direct and indirect effects across different noisy scenarios are presented in Table S6. TPR and FPR are shown in Table S7.

B.4 Simulation Scenario 4

The configuration was set with $p_1 = 5$ and $q_1 = 3$. We set M_3 to correlate with E_2, E_3, and E_4, but not with Y. The coefficients for mediators on Y are $(0.5, 0.8, 0)$, while all five exposure variables correlate with Y with

Table S4: Bias and standard deviation (in parentheses) using HDM-SCCA2 in estimating the direct and indirect effects under Scenario 2 (sample size $n = 1000$)

	Oracle	$N_e = 15$ $N_m = 30$	$N_e = 40$ $N_m = 25$	$N_e = 50$ $N_m = 70$	$N_e = 70$ $N_m = 100$	$N_e = 250$ $N_m = 200$	$N_e = 500$ $N_m = 500$
**Direct Effects**							
E1	-0.002 (0.008)	-0.011 (0.047)	-0.006 (0.045)	-0.010 (0.037)	-0.009 (0.034)	-0.011 (0.024)	-0.008 (0.024)
E2	0.004 (0.009)	-0.000 (0.047)	0.008 (0.045)	-0.002 (0.037)	-0.001 (0.034)	-0.019 (0.024)	-0.014 (0.024)
E3	0.006 (0.008)	-0.072 (0.047)	-0.072 (0.045)	-0.081 (0.037)	-0.079 (0.034)	-0.083 (0.024)	-0.076 (0.024)
E4	-0.001 (0.009)	-0.018 (0.047)	-0.018 (0.045)	-0.014 (0.037)	-0.013 (0.034)	-0.030 (0.024)	-0.026 (0.024)
E5	-0.009 (0.009)	0.099 (0.047)	0.098 (0.045)	0.111 (0.037)	0.116 (0.034)	0.147 (0.024)	0.158 (0.024)
**Indirect Effects**							
E1 M1	0.001 (0.009)	0.009 (0.039)	0.015 (0.042)	0.018 (0.043)	0.018 (0.043)	0.026 (0.043)	0.018 (0.041)
E2 M1	0.001 (0.009)	0.008 (0.039)	0.015 (0.042)	0.018 (0.043)	0.017 (0.043)	0.027 (0.043)	0.019 (0.041)
E2 M2	0.023 (0.009)	-0.018 (0.039)	-0.003 (0.042)	0.007 (0.043)	0.008 (0.043)	0.017 (0.043)	0.017 (0.041)
E3 M1	0.002 (0.009)	0.011 (0.039)	0.019 (0.042)	0.023 (0.043)	0.022 (0.043)	0.032 (0.043)	0.021 (0.041)
E3 M3	-0.000 (0.009)	-0.009 (0.039)	-0.017 (0.042)	-0.015 (0.043)	-0.011 (0.043)	0.003 (0.043)	-0.001 (0.041)
E3 M2	0.007 (0.009)	-0.014 (0.039)	0.004 (0.042)	0.017 (0.043)	0.023 (0.043)	0.041 (0.043)	0.040 (0.041)
E4 M2	0.006 (0.008)	-0.072 (0.039)	-0.048 (0.042)	-0.036 (0.043)	-0.035 (0.043)	-0.027 (0.043)	-0.029 (0.041)
E4 M3	-0.009 (0.009)	0.023 (0.039)	0.006 (0.042)	0.003 (0.043)	0.007 (0.043)	0.022 (0.043)	0.023 (0.041)
E5 M3	-0.003 (0.009)	-0.050 (0.039)	-0.073 (0.042)	-0.083 (0.043)	-0.079 (0.043)	-0.085 (0.043)	-0.088 (0.041)

Table S5: TPR and FPR for selected exposures and mediators using HDM-SCCA2 in simulation Scenario 2

n	N_e	N_m	TPR		FPR	
			Exposures	Mediators	Exposures	Mediators
1000	15	30	1.000	1.000	0.034	0.177
	40	25	0.998	1.000	0.035	0.265
	50	75	1.000	1.000	0.049	0.139
	70	100	1.000	1.000	0.043	0.121
	250	200	1.000	1.000	0.026	0.115
	500	500	1.000	1.000	0.014	0.068

coefficients $(0.15, 0.32, 0.28, 0.25, 0.2)$. The coefficients for E_i on M_i are set to 0.5, for E_i on M_{i+1} to 0.6, and

Table S6: Bias and standard deviation (in parentheses) of HDM-SCCA2 in estimating the direct and indirect effects under Scenario 3 (sample size n = 1000)

	Oracle	$N_e = 15$<br>$N_m = 30$	$N_e = 40$<br>$N_m = 25$	$N_e = 50$<br>$N_m = 70$	$N_e = 70$<br>$N_m = 100$	$N_e = 250$<br>$N_m = 200$	$N_e = 500$<br>$N_m = 500$
**Direct Effects**							
E1	-0.00<br>(0.01)	0.08<br>(0.07)	0.08<br>(0.07)	0.08<br>(0.08)	0.08<br>(0.07)	0.08<br>(0.07)	0.08<br>(0.07)
E2	0.00<br>(0.01)	-0.04<br>(0.07)	-0.05<br>(0.07)	-0.05<br>(0.08)	-0.05<br>(0.07)	-0.05<br>(0.07)	-0.05<br>(0.07)
E3	0.00<br>(0.01)	-0.04<br>(0.07)	-0.03<br>(0.07)	-0.02<br>(0.08)	-0.02<br>(0.07)	-0.02<br>(0.07)	-0.03<br>(0.07)
E4	-0.00<br>(0.01)	0.10<br>(0.07)	0.11<br>(0.07)	0.10<br>(0.08)	0.11<br>(0.07)	0.11<br>(0.07)	0.10<br>(0.07)
E5	-0.01<br>(0.01)	-0.11<br>(0.07)	-0.12<br>(0.07)	-0.12<br>(0.08)	-0.12<br>(0.07)	-0.12<br>(0.07)	-0.12<br>(0.07)
**Indirect Effects**							
E1 M1	-0.10<br>(0.03)	-0.11<br>(0.11)	-0.10<br>(0.11)	-0.11<br>(0.11)	-0.10<br>(0.11)	-0.10<br>(0.11)	-0.11<br>(0.11)
E2 M1	-0.11<br>(0.03)	-0.12<br>(0.11)	-0.12<br>(0.11)	-0.12<br>(0.11)	-0.12<br>(0.11)	-0.12<br>(0.11)	-0.12<br>(0.11)
E2 M2	-0.08<br>(0.03)	-0.11<br>(0.11)	-0.11<br>(0.11)	-0.11<br>(0.11)	-0.11<br>(0.11)	-0.11<br>(0.11)	-0.12<br>(0.11)
E3 M1	-0.09<br>(0.03)	-0.09<br>(0.11)	-0.09<br>(0.11)	-0.09<br>(0.11)	-0.09<br>(0.11)	-0.09<br>(0.11)	0.09<br>(0.11)
E3 M3	-0.08<br>(0.03)	-0.08<br>(0.11)	-0.08<br>(0.11)	-0.08<br>(0.11)	-0.08<br>(0.11)	-0.08<br>(0.11)	-0.08<br>(0.11)
E3 M2	-0.13<br>(0.03)	-0.15<br>(0.11)	-0.15<br>(0.11)	-0.15<br>(0.11)	-0.15<br>(0.11)	-0.15<br>(0.11)	-0.15<br>(0.11)
E4 M2	-0.10<br>(0.03)	-0.09<br>(0.11)	-0.09<br>(0.11)	-0.09<br>(0.11)	-0.09<br>(0.11)	-0.09<br>(0.11)	-0.09<br>(0.11)
E4 M3	-0.10<br>(0.03)	-0.10<br>(0.13)	-0.09<br>(0.11)	-0.09<br>(0.11)	-0.09<br>(0.11)	-0.09<br>(0.11)	-0.09<br>(0.11)
E5 M3	-0.07<br>(0.03)	-0.06<br>(0.11)	-0.06<br>(0.11)	-0.06<br>(0.11)	-0.05<br>(0.11)	-0.06<br>(0.11)	-0.06<br>(0.11)

Table S7: TPR and FPR for selected exposures and mediators using HDM-SCCA2 under Scenario 3

n	N_e	N_m	TPR		FPR	
			Exposures	Mediators	Exposures	Mediators
1000	15	30	1.00	1.00	0.00	0.00
	40	25	1.00	1.00	0.00	0.00
	50	75	1.00	1.00	0.00	0.00
	70	100	1.00	1.00	0.00	0.00
	300	200	1.00	1.00	0.00	0.00
	500	500	1.00	1.00	0.00	0.00

for E_i on M_{i+2} to 0.4. The results showing the bias of the estimates for both direct and indirect effects across

different noisy scenarios are presented in Table S8. TPR and FPR are shown in Table S9.

Table S8: Bias and standard deviation (in parentheses) using HDM-SCCA2 in estimating the direct and indirect effects under Scenario 4 (sample size n = 1000)

	Oracle	$N_e = 15$ $N_m = 30$	$N_e = 40$ $N_m = 25$	$N_e = 50$ $N_m = 70$	$N_e = 70$ $N_m = 100$	$N_e = 250$ $N_m = 200$	$N_e = 500$ $N_m = 500$
**Direct Effects**							
E1	-0.00 (0.01)	0.03 (0.07)	0.04 (0.07)	0.03 (0.08)	0.04 (0.08)	0.03 (0.08)	0.03 (0.08)
E2	0.00 (0.01)	0.00 (0.07)	0.01 (0.07)	0.01 (0.08)	0.01 (0.08)	0.00 (0.08)	0.00 (0.08)
E3	0.00 (0.01)	0.05 (0.07)	0.05 (0.07)	0.06 (0.08)	0.05 (0.08)	0.05 (0.08)	0.05 (0.08)
E4	-0.00 (0.01)	-0.00 (0.07)	-0.01 (0.07)	-0.01 (0.08)	-0.01 (0.08)	-0.00 (0.08)	-0.01 (0.08)
E5	-0.01 (0.01)	-0.06 (0.07)	-0.06 (0.07)	-0.06 (0.08)	-0.06 (0.08)	-0.06 (0.08)	-0.06 (0.08)
**Indirect Effects**							
E1 M1	-0.07 (0.02)	-0.11 (0.09)	-0.11 (0.09)	-0.11 (0.09)	-0.11 (0.09)	-0.11 (0.09)	-0.10 (0.09)
E2 M1	-0.08 (0.02)	-0.15 (0.09)	-0.15 (0.09)	-0.15 (0.09)	-0.16 (0.09)	-0.15 (0.09)	-0.14 (0.09)
E2 M2	-0.08 (0.02)	-0.16 (0.09)	-0.16 (0.09)	-0.16 (0.09)	-0.16 (0.09)	-0.16 (0.09)	-0.17 (0.09)
E3 M1	-0.06 (0.02)	-0.09 (0.09)	-0.09 (0.09)	-0.09 (0.09)	-0.10 (0.09)	-0.09 (0.09)	-0.09 (0.09)
E3 M3	0.00 (0.02)	0.00 (0.09)	0.00 (0.09)	0.00 (0.09)	0.00 (0.09)	0.00 (0.09)	0.00 (0.09)
E3 M2	-0.13 (0.02)	-0.20 (0.09)	-0.19 (0.09)	-0.19 (0.09)	-0.19 (0.09)	-0.20 (0.09)	-0.20 (0.09)
E4 M2	-0.10 (0.02)	-0.09 (0.09)	-0.09 (0.09)	-0.09 (0.09)	-0.09 (0.09)	-0.09 (0.09)	-0.10 (0.09)
E4 M3	0.00 (0.02)	0.00 (0.09)	0.00 (0.09)	0.00 (0.09)	0.00 (0.09)	0.00 (0.09)	0.00 (0.09)
E5 M3	0.00 (0.02)	0.00 (0.09)	0.00 (0.09)	0.00 (0.09)	0.00 (0.09)	0.00 (0.09)	0.00 (0.09)

Table S9: TPR and FPR for selected exposures and mediators using HDM-SCCA2 under Scenario 4

n	N_e	N_m	TPR		FPR	
			Exposures	Mediators	Exposures	Mediators
1000	15	30	1.00	1.00	0.00	0.00
	40	25	1.00	1.00	0.00	0.00
	50	75	1.00	1.00	0.00	0.00
	70	100	1.00	1.00	0.00	0.00
	300	200	1.00	1.00	0.00	0.00
	500	500	1.00	1.00	0.00	0.00

Appendix C Additional Simulation Studies on Tuning Parameter Selection and Error Variance Heterogeneity

Appendix C.1 Choice of Canonical Pair Threshold

In the HDM-SCCA framework, the number of retained canonical pairs c is chosen as the smallest value such that the selected pairs explain at least a prespecified proportion of the total *squared* canonical correlation. In the main analysis, we use a 70% threshold to balance information retention and model parsimony, retaining dominant signal while avoiding higher-order components that are more likely to capture noise.

To examine sensitivity to this choice, we conducted additional simulations under thresholds of 70%, 80%, and 90% (with $(N_e, N_m) = (15, 30)$). The results (Table S10) indicate that the threshold primarily affects the bias–variance trade-off. Lower thresholds tend to retain fewer components and may underfit in some settings, whereas higher thresholds include more components and exhibit increased variability. No single threshold consistently outperforms the others across all effects; instead, performance is comparable within the range of 70%–90%, suggesting that the method is not overly sensitive to this choice.

We also note that c is constrained by

$$c \leq \min\{\dim(\text{Exposure}), \dim(\text{Mediator})\},$$

and the regression step is performed on the reduced set of canonical variables. In all simulations, the selected c remained well below the sample size n, and we did not encounter cases with $c > n$, ensuring stable estimation.

Table S10: Comparison of Bias and Standard Deviation (SD) in Direct and Indirect Effects Across Thresholds

	Threshold = 0.7		Threshold = 0.8		Threshold = 0.9	
	Bias	(SD)	Bias	(SD)	Bias	(SD)
**Direct Effects**						
E1	-0.002	(0.067)	-0.053	(0.097)	-0.001	(0.054)
E2	0.019	(0.067)	-0.026	(0.097)	0.013	(0.054)
E3	-0.048	(0.067)	0.017	(0.097)	-0.034	(0.054)
E4	-0.014	(0.067)	-0.021	(0.097)	-0.004	(0.054)
E5	0.035	(0.067)	0.082	(0.097)	0.110	(0.054)
**Indirect Effects**						
E1 $\rightarrow$ M1	-0.051	(0.054)	0.029	(0.043)	0.033	(0.039)
E2 $\rightarrow$ M1	-0.042	(0.054)	0.019	(0.043)	0.021	(0.039)
E2 $\rightarrow$ M2	0.063	(0.054)	-0.004	(0.043)	0.008	(0.039)
E3 $\rightarrow$ M1	-0.121	(0.054)	0.058	(0.043)	0.043	(0.039)
E3 $\rightarrow$ M3	-0.099	(0.054)	-0.020	(0.043)	-0.044	(0.039)
E3 $\rightarrow$ M2	0.144	(0.054)	-0.010	(0.043)	-0.014	(0.039)
E4 $\rightarrow$ M2	0.117	(0.054)	-0.009	(0.043)	-0.001	(0.039)
E4 $\rightarrow$ M3	-0.079	(0.054)	-0.016	(0.043)	-0.025	(0.039)
E5 $\rightarrow$ M3	-0.053	(0.054)	-0.023	(0.043)	-0.063	(0.039)

Appendix C.2 Heterogeneous Mediator Error Variances

The primary simulations assume a common error variance across mediators to provide a controlled baseline. To better reflect realistic settings, we extended the simulations by allowing heterogeneous mediator error variances. Specifically, mediator errors were generated from a multivariate normal distribution with diagonal covariance matrix Σ_M, where the diagonal entries range from 0.2 to 1.2. The results (Table S10) are qualitatively consistent with those under homogeneous variance, with similar patterns in bias and variability for both direct and indirect effects. This suggests that the proposed method is robust to heterogeneity in mediator variability.

Appendix D Demographics for the Real Data

Table S11 summarizes the baseline demographic and clinical characteristics of the study population. A total of 114 participants were included in the analysis, with a mean age of 45.54 years (SD = 10.75) and a mean body mass index of 29.80 kg/m^2 (SD = 7.73). The cohort was predominantly male (60.5%), and the majority of participants identified as White (88.6%). Hispanic ethnicity was reported by 71.1% of subjects. Overall, the demographic

distribution reflects a relatively homogeneous study population, which reduces potential confounding due to population heterogeneity in the subsequent mediation analyses.

The chemicals and metabolites identified in the mediation analysis all had a detection rate of 1.00, indicating that nonzero measurements were observed for each participant. This complete detection across subjects minimizes concerns related to sparsity or zero inflation and supports the stability of downstream high-dimensional mediation modeling.

Table S11: Baseline Characteristics of the Study Population

**Characteristic**	**Level**	**Overall (N = 114)**
Age, years	Mean (SD)	45.54 (10.75)
BMI, kg/m^2	Mean (SD)	29.80 (7.73)
Sex	Male	69 (60.5%)
	Female	45 (39.5%)
Race	White	101 (88.6%)
	Black	10 (8.8%)
	Asian	1 (0.9%)
	Other	2 (1.8%)
Ethnicity	Hispanic	81 (71.1%)
	Non-Hispanic	33 (28.9%)
MELD Score	Mean(SD)	21.99 (6.52)
